# Tight associations between transcription promoter type and epigenetic variation in histone positioning and modification

**DOI:** 10.1186/1471-2164-12-416

**Published:** 2011-08-17

**Authors:** Tadasu Nozaki, Nozomu Yachie, Ryu Ogawa, Anton Kratz, Rintaro Saito, Masaru Tomita

**Affiliations:** 1 Institute for Advanced Biosciences, Keio University, Tsuruoka, 997-0017, Japan; 2 Systems Biology Program, Department of Environment and Information Studies, Keio University, Fujisawa, 252-8520, Japan; 3 Department of Biological Chemistry and Molecular Pharmacology, Harvard Medical School, Boston, MA 02445, USA; 4 Systems Biology Program, Graduate School of Media and Governance, Keio University, Fujisawa, 252-8520, Japan; 5 Systems Biology Program, Department of Environment and Information Studies, Keio University, Fujisawa, Kanagawa 252-8520, Japan; 6 Donnelly Centre of Cellular and Biomolecular Research, University of Toronto, Toronto, ON M5S-3E1, Canada; 7 Department of Medicine, University of California, San Diego, La Jolla, CA 92093, USA; 8 Omics Science Center, RIKEN Yokohama Institute, 1-7-22 Suehiro-cho, Tsurumi-ku, Yokohama, Kanagawa 230-0045 Japan

## Abstract

**Background:**

Transcription promoters are fundamental genomic *cis*-elements controlling gene expression. They can be classified into two types by the degree of imprecision of their transcription start sites: peak promoters, which initiate transcription from a narrow genomic region; and broad promoters, which initiate transcription from a wide-ranging region. Eukaryotic transcription initiation is suggested to be associated with the genomic positions and modifications of nucleosomes. For instance, it has been recently shown that histone with H3K9 acetylation (H3K9ac) is more likely to be distributed around broad promoters rather than peak promoters; it can thus be inferred that there is an association between histone H3K9 and promoter architecture.

**Results:**

Here, we performed a systematic analysis of transcription promoters and gene expression, as well as of epigenetic histone behaviors, including genomic position, stability within the chromatin, and several modifications. We found that, in humans, broad promoters, but not peak promoters, generally had significant associations with nucleosome positioning and modification. Specifically, around broad promoters histones were highly distributed and aligned in an orderly fashion. This feature was more evident with histones that were methylated or acetylated; moreover, the nucleosome positions around the broad promoters were more stable than those around the peak ones. More strikingly, the overall expression levels of genes associated with broad promoters (but not peak promoters) with modified histones were significantly higher than the levels of genes associated with broad promoters with unmodified histones.

**Conclusion:**

These results shed light on how epigenetic regulatory networks of histone modifications are associated with promoter architecture.

## Background

Recent progress in high-throughput technologies has made it possible to collect a variety of "omics" data on transcripts and on the epigenetic behaviors of the histones that are often associated with these transcripts [1-5].

Cap analysis of gene expression (CAGE) is a high-throughput method that enables large-scale identification of transcription start sites (TSSs) of eukaryotic species. This method measures gene expression levels simultaneously with TSS identification by counting the sequenced 5' ends of full-length cDNAs, termed CAGE tags [2,6]. With the development of deep sequencing methods, more high-throughput, and high resolution "tag depth" measurements have become available (DeepCAGE, nanoCAGE and CAGEscan) [1,7]. Such recent whole-cell-level pictures of quantitative transcriptomes have revealed the complex transcriptional network of mammalian species [1,2,6]. According to recent CAGE-based analyses of human TSSs, the human "promotome" can be classified into two types of promoters by the degree of imprecision of their transcription initiation sites [8]. One is the peak promoter, which initiates transcription strictly from a narrow genomic region (within a distance of 1-4 bp), and the other is the broad promoter, which initiates transcription from wide-ranging positions (> 4 bp) [8,9]. The peak promoters are suggested to be closely associated with the presence of the TATA box (which enables proper control of gene expression by binding with transcription factors) and with tissue-specific gene expression. The broad promoters have been observed in the presence of CpG islands and drive relatively ubiquitous expression of the genes they control [8,10-12]. The CpG-rich broad promoters are considered evolutionarily new and more likely to be controlled by epigenetic mechanisms, including DNA methylation and sense-antisense regulation, than the peak promoters [8,11,13]. These differences between broad and peak promoters raise questions of how these promoter types are associated with chromosomal structures and modifications and of how their difference confers cellular function.

In eukaryotic species, chromosomal DNAs is packed into nucleosomes, each of which comprises approximately 147 base pairs wrapped around a histone protein octamer consisting of two copies of each of the four core histones, H2A, H2B, H3, and H4 [14,15]. Two biologically important aspects of these histones are their positions and modifications, and it has been shown that these factors regulate transcription initiation [16-18]. Several methodologies have rapidly been developed for high-throughput identification of histone positions and modifications. ChIP-chip identifies the histone-binding positions of genomic DNA by using a combination of chromatin immunoprecipitation and tiling array [19]. Although ChIP-chip used to be a widely-used method, today, with the growing demand to develop high-throughput sequencing, the ChIP-Seq method has been developed as a promising alternative to the tiling array-based approach in analyzing genome-wide nucleosome positioning [20,21]. These methodologies have revealed several insights into the intertwining of gene expression with nucleosome position and histone modification. For example, the degree of eviction of nucleosomes from the upstream regions of TSSs is correlated with gene expression patterns in yeasts [19,22] and humans [4,23,24]. Moreover, the methylated histone H3 at lysine 4 (H3K4me1, 2, and 3) and acetylated histone H3 at lysine 9 (H3K9ac), located around TSSs, are linked to gene activation [3,25-28], whereas H3K27me3 and H3K9me3 are linked to gene repression [3,27,29,30]. These modifications and related gene regulatory behaviors support the "histone code" hypothesis [28], i.e. that multiple histone modifications specify unique downstream functions. However, the detailed mechanisms underlying transcriptional regulation by these histone behaviors are still obscure.

H3K9ac has recently been frequently observed around broad promoters [9]. This implies that histone behavior is associated with promoter architecture, although this association has so far been found only in the case of H3K9ac, and the extent of such associations is unclear. In this study, we systematically analyzed the relationships between histone behaviors and promoter architecture types by using information about (1) modified/unmodified histones; (2) their genomic positions relative to TSSs; (3) their positional stabilities on the genome under two cellular conditions; and (4) gene expression. The results showed that promoter architecture type and gene expression are tightly associated with the modification pattern and genomic positional stability of the histones forming nucleosomes. They provide new insights into the epigenetic mechanisms of transcriptional regulation in terms of histone behavior.

## Results

### Promoter architecture and nucleosome positioning

**Figure 1:**
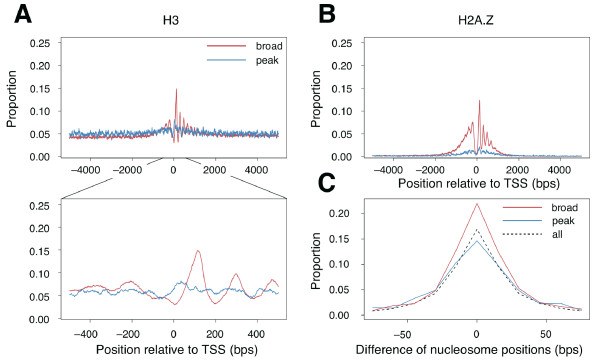
**Distributions of nucleosome positions around transcription start sites (TSSs)**. (A) Distributions of the central positions of histone H3 around broad and peak promoters. The *x*-axis shows genomic positions with respect to TSSs (from -5 kb to 5 kb, upper panel; and from -500 bp to 500 bp, lower panel). The central positions of nucleosomes are defined as the positions from -15 bp to 15 bp with respect to the center of the nucleosome. (B) Distributions of nucleosomes containing the histone variant H2A.Z around TSSs (from -5 kb to 5 kb). H2A.Z around TSSs associated with broad promoters are highly enriched, unlike those associated with peak promoters. (C) Distributions of minimum distances from each of the nucleosomes in human resting T cells compared with those in activated T cells. The *x*-axis shows the minimum distances and the *y*-axis shows the proportions of nucleosomes with the specified minimum distances. Proportions within every 15 bp were averaged. Minimum distances were calculated for all nucleosomes on the genome (dashed line), for those associated with broad promoters (red line), and for those associated with peak promoters (blue line).

We first focused on differences in nucleosome distribution around the two different types of transcription promoters (i.e. peak and broad promoters). We used human promoter positions for which information about the degree of transcription start imprecision had been obtained in a previous study [9], as well as nucleosome positions defined as the genomic positions of histone H3 proteins in the resting condition in human CD4+ T-cells [4]. We mapped them on human genomic sequences. (See Methods for details of data manipulations.) We then calculated the ratio of nucleosomes located at each genomic position relative to each peak and broad promoter. We found that the nucleosome positions associated with broad promoters had markedly aligned and periodic patterns compared with those of peak promoters (Figure 1A). More strikingly, only in broad promoters, the first nucleosomes immediately downstream of the promoter were likely to be located in similar positions and those immediately upstream of the promoter were depleted (see the magnified view in Figure 1A). This was contrary to our expectation; previous studies have reported that, in general, nucleosomes are distributed evenly around the promoter region [31,32]. We had therefore expected that the nucleosome positions would be spread around the broad promoter and well aligned around the peak promoter, because TSSs are widely spread in the broad promoter region but narrowly spread in the peak promoter region. However, our results show that the broad promoter was specifically associated with a more aligned pattern of nucleosomes than the peak promoter.

H2A.Z is a histone variant of H2A that is highly conserved among lower and higher eukaryotes. Enrichment of H2A.Z around the promoter region has been also reported in yeast [33] and humans [34]. In terms of promoter architecture, we performed a similar analysis to the one of H3 shown in Figure 1A of the positions of human nucleosomes harboring the histone variant H2A.Z in human resting CD4+ T-cells [3]. H2A.Z was highly enriched around broad promoters but not peak promoters (Figure 1B). For example, the statistical significance of the enrichment was *P *< 1.0 × 10-25 (chi-squared test) for positions +100 to +130 with respect to the TSS. Moreover, the distribution patterns of H2A.Z were similar to those of H3; the positions of H2A.Z were markedly aligned around broad promoters but not around peak promoters.

### Accessibility of transcription factor Sp1

The two promoter architectures are associated with characteristic sequence contexts: the peak promoter is located close to a TATA box and the broad promoter close to CpG islands [8]. Using the genomic positions of putative TATA-box sites predicted by a position-specific weight matrix and the positions of CpG islands obtained from the UCSC Genome Browser database [35,36], we confirmed that TATA boxes were overrepresented in peak promoters and that broad promoters were highly associated with the presence of CpG islands (Additional file 1, Figure S1).

It is possible that the aligned patterns of nucleosome positions around broad promoters are due to the accessibility of transcription factors to DNA. For instance, in the absence of the TATA box, the ubiquitous transcription factor Sp1 can recruit TATA-binding proteins to initiate transcription [37]. It has already been reported that consensus Sp1 sites with high overall GC contents are overrepresented among broad promoters, and the positions of these sites for individual transcription units are less precise than those of TATA boxes [8]. Consequently, we investigated the possibility that the nucleosomes around a broad promoter align in a more orderly fashion than those around the peak promoter because of the need to create a nucleosome-free region upstream of the TSS to confer DNA accessibility of transcription factor proteins. We superimposed the distribution of putative Sp1 sites [1] around broad promoters onto that of the nucleosome positions (see Methods), and we observed increased proportions of Sp1 sites about -50 bp upstream of the broad promoter, where the nucleosome distribution was markedly depleted (Additional file 2, Figure S2). We conducted the same analysis for peak promoters. The inverse relationship between Sp1 site and nucleosome abundance around the broad promoter was much higher than that around the peak promoter, suggesting the plausibility of the DNA accessibility model. Furthermore, we conducted a similar analysis for the binding sites of two other transcription factors, PU.1 and MAZ, as a previous study (FANTOM4) had analyzed the binding sites of these two factors in detail [1]. The binding sites of both PU.1 and MAZ were distributed on nucleosome-free regions around broad promoters, whereas no such trends were observed around peak promoters (Additional file 3, Figure S3). These results support the strong connection between the nucleosome-free region and the accessibility of transcription factors, which was specific to broad promoters.

### Positional stability of nucleosomes around broad promoters

If nucleosome positioning around broad promoters confers DNA accessibility for the binding of transcription factors, then the nucleosome positions around broad promoters should be more stable throughout different cellular conditions than those around peak promoters, because broad promoters are usually associated with ubiquitously expressed gene (in contrast, peak promoters are associated with tissue- and condition-specific expressed gene) [8,10-12] and the genomic positions of transcription factor binding sites are fixed. We analyzed the positional stability of nucleosomes located within positions +1 to +200 with respect to each promoter under "resting" and "activated" conditions of human CD4+ T-cells [4] (see Methods). For each nucleosome position in the resting condition, we calculated the distance to the nearest nucleosome position in the activated condition in order to assess the positional stabilities of single nucleosomes under the two different cellular conditions. The overall minimum distance was markedly shorter for broad promoter-associated nucleosomes than for peak promoter-associated ones (Figure 1C). In fact, the average absolute minimum distance in the case of the broad promoter (20.70 bp) was significantly shorter than that for the peak promoter (25.08 bp) (*P *= 4.83 × 10^-7^; *t*-test. Note that we did not take into account nucleosomes for which a minimum distance longer than 100 bp was found between the two conditions, because these were more likely to be different or neighboring nucleosomes rather than those that moved along the DNA with the change in conditions.). These results demonstrated that the positions of nucleosomes around the broad promoters were more stable than those of nucleosomes around the peak promoters.

### Distribution of nucleosomes containing modified histones

**Figure 2:**
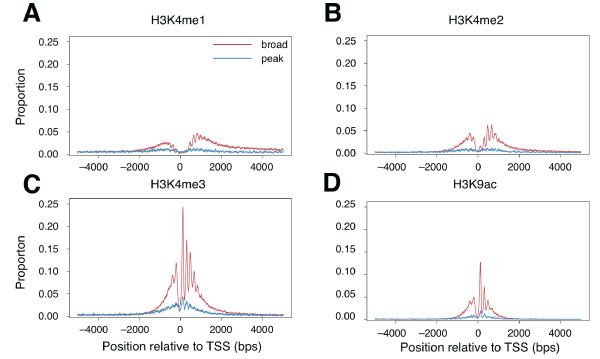
**Distribution of modified histones around transcription start sites (TSSs)**. Distributions of nucleosomes containing methylated and acetylated histones. (A) H3K4me1, (B) H3K4me2, and (C) H3K4me3 and (D) H3K9ac around TSSs are shown. All of the modified histones were highly enriched around the TSSs associated with broad promoters, unlike those associated with peak promoters. The *x*-axis shows the genomic positions with respect to the TSSs (from -5 kb to 5 kb).

**Figure 3:**
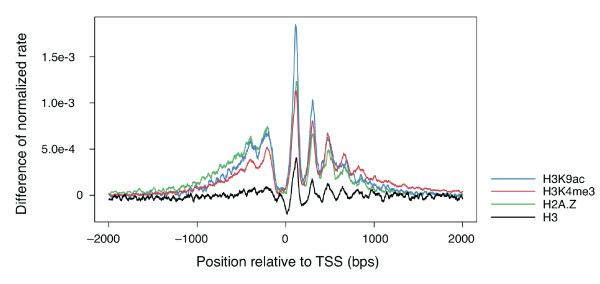
**Relative abundance in histone distributions**. Normalized differences in histone distributions (H3, H3K4me3, H3K9ac, and H2A.Z) between broad and peak promoters (from -2 kb to 2 kb) at each position are shown. The *y*-axis shows the normalized differences in histone distributions between broad and peak promoters. H3K4me3, H3K9ac, and H2A.Z had larger differences than H3.

It has been suggested that not only nucleosome position, but also nucleosomal histone modification, can regulate transcription [3,25-27]. For instance, histone methylation is associated with either gene activation or repression, depending on the methylation site and state on the histone protein; in particular, methylation of histone H3 (H3K4me1, -2, and -3) in nucleosomes around the transcription promoter are well known to regulate gene expression [3,25-28]. To investigate the differences in positional distribution of nucleosomes containing methylated histones around the two different types of promoter, we obtained nucleosome positions corresponding to each of three methylation types (H3K4me1, 2, and 3) in human CD4+ T cells from a previous study [3], and we mapped these onto genomic sequences with the broad and peak promoter positions. Similar to the result for histone H3, nucleosomes having H3K4me1, -2, and -3 were all highly enriched and well aligned around broad promoters, whereas they were depleted around peak promoter regions (Figure 2A-C). However, the alignment pattern of nucleosome positions differed depending on the type of methylation. Within the region downstream of the broad promoter, the first frequency peak of nucleosomes having H3K4me1 and 2 occurred in the +700 to +730 region (Figure 2A and 2B), whereas those having H3K4me3 occurred in the +100 to +130 region (Figure 2C; this was similar to the result for histone H3, perhaps because the majority of H3K4 were trimethylated). For each methylation type, the difference in frequency of occurrence of nucleosomes with each type of modified histone in these regions between the peak and broad promoters was significant (*P *< 1.0 × 10-10 for H3K4me1 and 2, and *P *< 1.0 × 10-50 for H3K4me3; chi-squared test). Note that the values on the *y*-axes in Figure 2 are not influenced by the absolute numbers of nucleosomes in each type of promoter, as they indicate the proportion of nucleosome-harboring TSSs for each type of TSS. In addition to methylation, acetylation may control gene expression [3,25-28]. We further analyzed nucleosome positioning corresponding to histone acetylation (H3K9ac) in human CD4+ T cells and observed results similar to those for H3K4me3 (*P *< 1.0 × 10-50 for +100 to +130 region; chi-squared test; Figure 2D). For each of H3, H2A.Z, H3K4me3, and H3K9ac, we estimated the abundance of nucleosomes associated with peak promoters relative to that of nucleosomes associated with broad promoters (Figure 3; see Methods). Compared with nucleosomes carrying histone H3, the relative abundances of nucleosomes carrying the modified histones or the histone variant were large, suggesting that the presence of histone modifications or a histone variant was highly associated with the broad promoter but not the peak promoter.

### Analysis of another genomic element that potentially influences histone behavior

**Figure 4:**
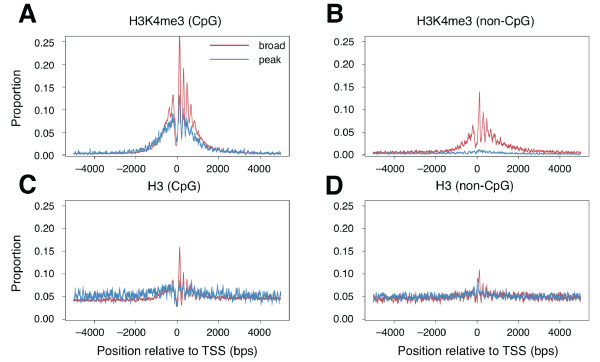
**Distributions of nucleosomes around transcription start sites (TSSs) with and without CpG islands**. Distributions of nucleosomes containing H3K4me3 (A, B) and H3 (C, D) around broad and peak promoters are shown. The analyses were conducted separately for TSSs that were associated with CpG islands (A, C) and those that were not (B, D). Broad promoters had aligned patterns of nucleosomes containing H3 and H3K4me3, regardless of the existence of CpG islands, and were enriched in H3K4me3. In contrast, peak promoters had little alignment of the H3 pattern, regardless of the presence of CpG islands. The proportion of nucleosomes containing H3K4me3 associated with peak promoters was lower than that associated with broad promoters, particularly in the absence of CpG islands.

Methylation of CpG islands is tightly associated with the expression of downstream genes; a number of studies have therefore been conducted to analyze CpG islands at a genome-wide level [38,39]. As described above, broad promoters are strongly associated with CpG islands (Additional file 1, Figure S1). Therefore, it is possible that the enrichment of histone modifications and histone variants in the broad promoter region is derived merely from the effect of CpG islands and is independent of promoter architecture. In fact, it has been shown that promoters with many CpG islands are more likely to harbor modified histones than promoters with fewer CpG islands [40]. To address this issue, we analyzed the positions of nucleosomes having histone H3 and those having H3K4me3 around broad and peak promoters with and without CpG islands (Figure 4). We found that, in the case where promoters were associated with CpG islands, nucleosomes with histone H3K4me3 were likely to be well aligned even around peak promoters. However, broad promoter-associated nucleosomes were significantly more enriched than peak promoter-associated nucleosomes, especially in the region downstream of the promoter (Figure 4A; *P *< 1.0 × 10-16 for +100 to +130 region; chi-squared test). (Note, however, that the set of "peak promoters" used in this study may have included "broad promoters," and that this may have affected the highly aligned nature of H3K4me3 around "peak promoters." This was because the definition of promoter architecture thus far was whether there was a cluster of TSSs located within a narrow genomic region or whether the TSSs were dispersed, and low TSS coverage increased the possibility of promoters being classified as "peak promoters".)

In contrast, when we focused only on promoters without CpG islands, nucleosomes having H3K4me3 were well aligned and enriched only around broad promoters (Figure 4B); the difference in the frequencies of downstream nucleosomes (from +100 to 130) potentially resulting from the difference in the alignment were significant (*P *< 1.0 × 10-56, chi-squared test). Broad promoters with CpG islands had an aligned pattern of nucleosomes carrying H3, whereas no clear alignment was observed for peak promoters (Figure 4C). Broad promoters without CpG islands still showed an aligned pattern of nucleosomes having H3 (although the pattern was less clear than in those with CpG islands), whereas peak promoters had little alignment in the pattern (Figure 4D). These results show that the enrichment of nucleosomes having certain histones around a broad promoter is independent of the existence of CpG islands.

### Effect of histone modification on gene expression

**Figure 5:**
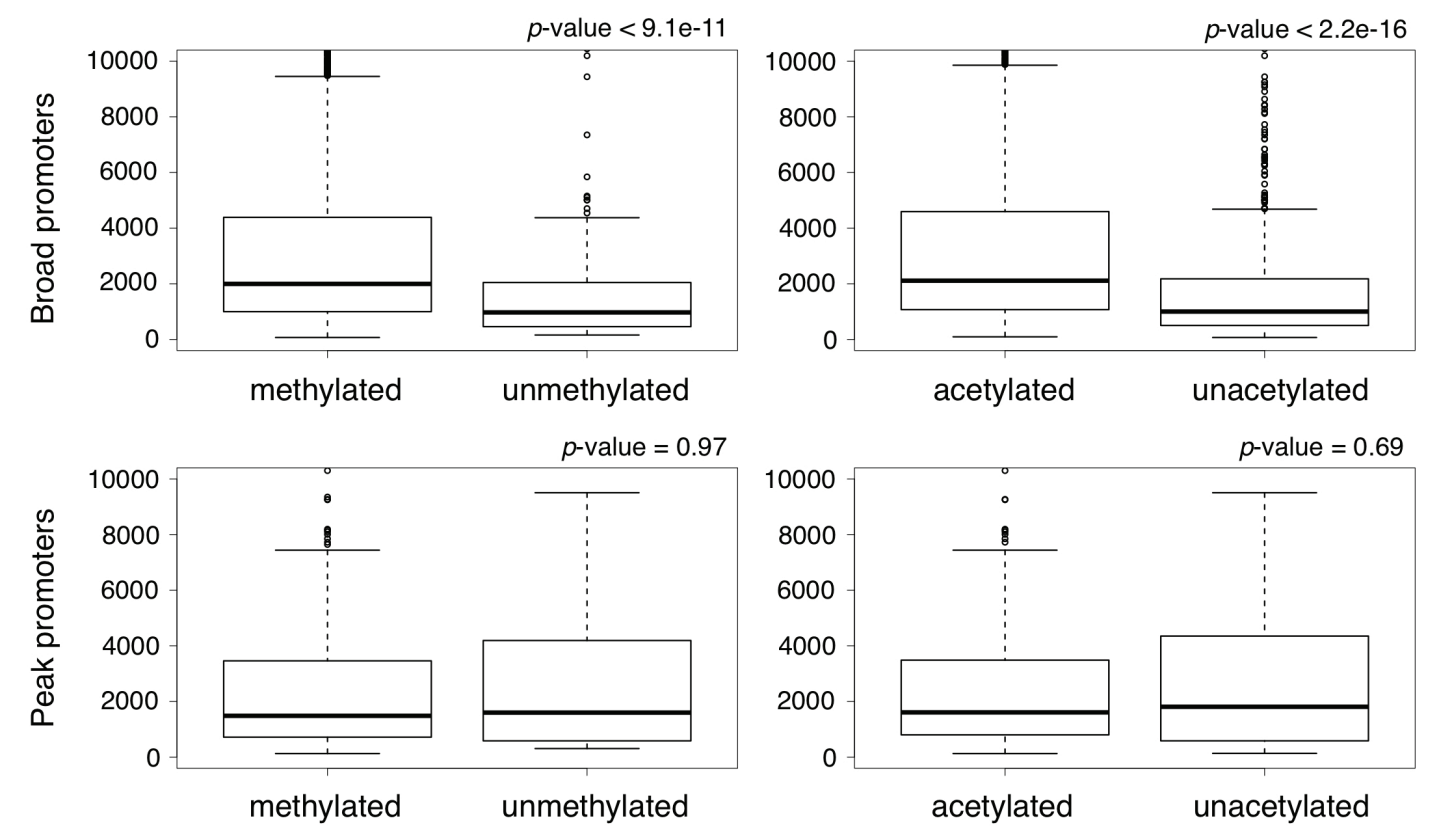
**Box plots of gene expression in human resting CD4+ T cells**. The box plots represent the distributions of gene expression levels. Distributions of the four groups of genes are drawn separately, i.e. those with broad or peak promoters, each of which was further associated with modified histones in activated cells or with unmodified histones. The *y*-axis shows the microarray intensities of the gene sets in each category.

To explore whether histone modification around the promoter affects gene expression, we analyzed the difference in expression levels of RNAs transcribed from peak and broad promoters in terms of the existence of modified/unmodified histones in their surrounding regions. We compared data sets of methylated/unmethylated histones and acetylated/unacetylated histones measured under resting conditions in human CD4+ T cells [3,5]. Gene expression data for resting CD4+ T cells were obtained from a previous study [4]; we used only those genes for which the expression levels had been measured. We classified promoters having at least one methylated/acetylated histone within the region from -500 to +500 as "promoters with methylated/acetylated histones" and all others as ones with unmethylated/unacetylated histones (see Methods). Expression levels of genes 'associated with broad promoters that had methylated histones were significantly higher than those of genes associated with broad promoters with unmethylated histones (*P *< 9.1 × 10-11, U-test; Figure 5). Conversely, the expression levels of genes associated with peak promoters having only unmethylated histones were as high as those of genes associated with peak promoters with methylated histones, and thus no significant difference was observed (*P *= 0.97, U-test; Figure 5). Likewise, in the comparison between acetylated and unacetylated histones, the expression levels of genes associated with broad promoters that had acetylated histones were significantly higher than those of genes associated with broad promoters with no acetylated histones (*P *< 2.2 × 10-16, U-test; Figure 5), but the expression levels of genes associated with peak promoters that had acetylated histones did not differ markedly from those of genes associated with peak promoters with only unacetylated histones (*P *= 0.69, U-test; Figure 5). These results suggest that the regulation of gene expression levels by histone modification is specific to broad promoter-associated genes.

## Discussion

We analyzed the global landscape of epigenetic relationships between histone modifications and transcription initiation by investigating genome-wide ChIP-Seq data and DeepCAGE data. The results presented here show differences in the architecture of the broad and peak promoters that regulate gene expression. Especially, we revealed that the broad promoters were strongly associated with histones immediately downstream of the TSS and they were frequently modified, presumably to regulate gene expression levels.

In previous studies, aligned patterns of nucleosome positions around TSSs have been identified in yeasts and humans [22,31,32,41]. However, we confirmed this alignment only for regions around TSSs derived from broad promoters, not for those around TSSs derived from peak promoters. Broad promoters have an aligned pattern of nucleosome positions around TSSs and have large nucleosome-free regions immediately upstream of TSSs. Studies in yeasts have validated the model of "open promoters," which have large, nucleosome-free regions immediately upstream of the TSS and are often associated with TATA-less promoters and poly (dA:dT)-rich tracts, the sequences of which are unbendable and unstable for histone binding [42]. The broad promoter characteristics that we found in humans are consistent with this model, because in humans the sequence patterns in CpG islands located upstream of TSSs, in contrast to the yeast poly (dA:dT)-rich tracts, have been shown to be unstable [31].

Our data indicate that the nucleosomes that are immediately downstream of TSSs and associated with broad promoters are positioned in specific regions. We suggest that broad promoters have these aligned patterns of nucleosome positions around TSSs because the nucleosome position has a stronger impact on broad promotors than on peak promoters on the determination of TSSs by transcription factors in the cell.

As an example of transcription factors that target broad promoters, we investigated the Sp1 binding sites around TSSs. Sp1 recognizes binding region of DNA via its zinc finger domain whereas TBP recognizes TATA box via its DNA binding domain. Sp1 binding sites were enriched in the regions upstream of TSSs corresponding to the nucleosome-free regions. We observed similar tendencies for the binding sites of two transcription factors, PU.1 and MAZ. Although biological experiments are necessary to investigate molecular mechanism behind this observation, we speculate that the nucleosome-free regions serve as "landing sites" for transcription factors, including Sp1, which have less precise binding motifs (which are overrepresented among broad promoters) than the TATA box [43-45].

In addition to histone H3, we also analyzed the positions of the histone H2A variant H2A.Z, which is enriched around TSSs [46], and we obtained similar results. In contrast, peak promoters did not have aligned patterns of nucleosome positions. One might suspect that the observation is due to high expression of genes associated with broad promoters, and low expression of those associated with peak promoters. However even after we limited the analysis to broad and peak promoters both of which are associated with highly expressed genes, we still observed the preferences of H3 for broad promoters (region 100-130 bp with respect to TSSs) compared to peak promoters (*P *< 1.0 × 10^-9^, chi-squared test, data not shown). Although TSSs for TATA promoters are often fixed to single positions, our results suggest that such strictly controlled positions of TSSs are not regulated by nucleosome position. However, there is some evidence that the nucleosomes around TATA promoters have regulatory roles in gene expression. In yeasts, the TATA promoter is one type of "covered promoter," and expression of the genes associated with such promoters is more likely to be inhibited by the presence of nucleosomes than expression of the genes associated with "open promoters," which are located in nucleosome-free regions [42]; in covered promoters, nucleosomes often cover transcription factor binding sites to repress the expression of downstream genes. It is also possible that, in humans, peak promoters associated with the TATA box belong to one type of "covered promoter" where the expression of downstream genes is repressed by the presence of nucleosomes. Therefore, we speculate that transcription factor binding is controlled by nucleosome position in the case of peak promoters.

In our analysis of epigenetic control by histone modification, we uncovered an difference between broad and peak promoters. H3K4me1, -2, and -3 and H3K9ac, which are associated with gene activation, were more highly enriched around TSSs associated with broad promoters than around those associated with peak promoters. Thus broad promoters appeared to be under stronger epigenetic control than peak promoters. We found a trend that further supported this hypothesis: the expression levels of genes associated with broad promoters that had modified histones had higher expression levels than genes associated with broad promoters without modified histones. In contrast, peak promoters appeared to be under weaker epigenetic control, because far fewer of them harbored modified histones. Furthermore, there were no significant differences in the expression levels of genes associated with peak promoters that harbored or did not harbor modified histones.

It has been shown that promoters with many CpG islands are more likely to harbor modified histones than promoters with fewer CpG islands [40]. However, even after we limited our analysis to promoters having CpG islands, number of broad promoters harboring H3K4me3 was still statistically higher than that of peak promoters. Even more remarkable differences were observed after we limited our analysis to promoters without CpG islands. Although these results may depend on the dataset of CpG islands we used, enrichment of H3K4me3 in downstream region (+100 to +130-bp) of broad promoters were still observed in the analysis using different dataset of CpG islands [47] (*P *value of < 1.0 × 10^-20 ^for CpG-related genes, *P *value of < 1.0 × 10^-30 ^for CpG-unrelated genes).

Genes associated with broad promoters tend to be expressed ubiquitously, whereas those associated with peak promoters are likely to be expressed in specific tissues and may show low expression levels in most tissue types [8]. Therefore, if high levels of gene expression are directly associated with histone modifications around TSSs, then we may observe spurious correlations between promoter type and histone modification. In fact, H3K4me3 is known to upregulate the expression of downstream genes. We therefore compared the distribution patterns of nucleosomes containing H3K4me3 around broad and peak promoters in cases where the downstream genes showed similar expression levels (Additional file 4, Figure S4). We found that the broad promoters also harbored more nucleosomes containing H3K4me3 in cases where the downstream genes showed similar expression levels (data not shown); the difference in the distributions of H3K4me3 around the broad and peak promoters was statistically significant (all positions from +100 to +130 showed significant differences; *P *< 1.0 × 10^-3^, chi-squared test), suggesting that promoter type was indeed associated with differences in epigenetic regulation by histone modifications.

Peak promoters containing the TATA box are regulated at their transcription initiation step, generally by the assembly of a pre-initiation complex with three additional components: the TATA-associated factors, the so-called mediator complexes, and positive and negative cofactors. We presume that peak promoters containing no TATA box are regulated in a similar way. This transcription system is widely used in various species, and our results suggest that it is unlikely to use epigenetic controls. Thus, broad and peak promoters have distinct systems to regulate gene expression.

Throughout this work, we employed widely-accepted definition of peak promoters, i.e. those which initiate transcription within the range of 4 bps. Changing this threshold to 10 bp did not have much effect on the distribution patterns of nucleosomes around broad and peak promoters as shown by Pearson's correlation coefficients between histone distribution pattern around broad promoters (-5000 to 5000 bps with respect to TSS) defined by > 4 bps threshold and that defined by > 10 bps threshold. For H3 distribution patterns, correlation coefficients were 0.99 and 0.94 for broad and peak promoters, respectively. For H3K4me3 distribution patterns, the correlation coefficients were 0.99 for both broad and peak promoters. These results suggest the robustness of the relationships between the imprecision of TSS and patterns of histone distributions.

TATA boxes are used in a wide range of organisms, including prokaryotes, and are thought to be part of an ancient transcriptional system. In contrast, broad promoters are thought to be newly evolved [8] and have incorporated histone modification systems. Our results showed that peak promoters, which are frequently associated with such ancient TATA boxes, have not incorporated histone modification systems.

## Conclusions

By using a computational approach, we discovered the general relationships between the two types of promoter architecture and histone behavior, including positioning and modification. We first showed that the positions of histones around broad promoters were highly aligned and stable compared with those around peak promoters. Furthermore, we suggest that marked numbers of transcription initiations related to broad promoters are under the control of histone methylation and acetylation and are associated with gene expression level, whereas this is not the case with peak promoters. These results indicate that the expression of genes associated with broad promoters, but not peak promoters, is highly associated with histone position and modification. We believe that our study is a step in uncovering the general mechanisms underlying transcriptional systems and inferring how these systems have evolved. This should eventually help us to understand the complexity of mammalian transcription.

## Methods

### Nucleosome position detection and dataset

Nucleosome-resolution (MNase digestion) ChIP-Seq Solexa tags for histone H3 were obtained by [4]. The genomic positions of the methylated histones and the histone variant H2A.Z were obtained from [3], and those of acetylated histones were from the study by [5]. All of these data were obtained in human resting CD4+ T cells. To determine the genomic positions of nucleosomes according to the ChIP-Seq data, we used the software published in [48]. Human genome hg18 was used.

### Transcription start site detection and dataset

TSSs were detected by DeepCAGE data obtained by the FANTOM 4 project [1]; 10,971 TSSs of broad promoters and 3621 TSSs of peak promoters were detected by applying the methods used in FANTOM 3 [8,9] to the FANTOM 4 dataset [9]. We used only those promoters for which the corresponding probes were clustered on the genome (level 3 promoters; [1,9]), and for each promoter the neighboring position that had the highest density of overlapping CAGE tags was determined as the position of the TSS. Promoters containing TATA boxes within 50 bp upstream of TSSs were determined by using position-specific weight matrices from JASPAR4 [49] (with a confidence score of more than 75%), and promoters containing CpG islands within 200 bp upstream of TSSs were obtained from the UCSC Genome Browser database (http://genome.ucsc.edu/). Alternative dataset of CpG islands were obtained from [47].

### Distribution of nucleosome positions around TSSs

As described above, the genomic positions of nucleosomes as well as TSSs for both broad and peak promoters were determined. The distributions of nucleosomes within the genomic regions from -5 kb to 5 kb with respect to TSSs were calculated by dividing the number of nucleosomes at each position by the number of TSSs. Genomic positions from -15 bp to 15 bp with respect to the central positions of the nucleosomes were assumed as the genomic positions where nucleosomes existed. The distributions of nucleosomes near broad and peak promoters were calculated separately.

### Distribution of Sp1 binding sites and other transcription factor binding sites

Sp1, MAZ, and PU.1 binding sites were obtained from FANTOM 4 (http://fantom.gsc.riken.jp/4/download/GenomeBrowser/hg18/TFBS_CAGE/allsites_cage_tfbs_feb09_latest.gff.gz) [1]. The distributions of these transcription factor binding sites around TSSs (from -500 bp to 500 bp) were calculated by dividing the number of these sites for each position by the number of TSSs used for the analysis.

### Stability of nucleosome positions under different cellular conditions

We compared the nucleosome positions obtained in human resting CD4+ T cells with those obtained in human activated CD4+ T cells. We calculated the minimum distance between each nucleosome in resting T cells and the closest nucleosome in activated cells. This distance was considered to denote how far each nucleosome moved along the genome in response to the change in cellular condition (from resting to activated). The distributions of these distances were calculated by dividing the number of nucleosomes that moved specified distances (from × bp to × + 15 bp) by the total number of nucleosomes. The average absolute minimum distance between each nucleosome in resting T cells and the closest nucleosome in activated cells was also calculated.

### Relative abundances of peak promoters and broad promoters

The abundance of peak promoters relative to that of broad promoters at position *j *was calculated by (*B_j _*- *P_j_*)/Σ*_i_B_i_*, where *B_j _*and *P_j _*denote the proportions of nucleosomes at position *j *for broad and peak promoters, and Σ*_i_B_i _*denotes the sum of proportions of nucleosomes around the TSS (from -2000 bp to 2000 bp), respectively.

### Gene expression in human resting CD4+ T cells

The gene expression profile in human resting CD4+ T cells was obtained from the Gene Expression Omnibus (GSE10437) [4]. We used genes (total number 8007, with 7591 associated with broad promoters and 416 associated with peak promoters) annotated with Entrez gene IDs in FANTOM 4 and with expression present in Present/Absent calls generated by the Affymetrix microarray platform. Nineteen types of methylated histones and 18 types of acetylated histones obtained in CD4+ T cells were used [3,5]. Acetylated histones located around TSSs are linked only to gene activation. To investigate the upregulation of genes associated with histone acetylation and their dependence on promoter type, we made two groups of histones: one having modified histones (18 types of acetylated histone) around TSSs (from -500 bp to 500 bp) and the other having no modified histones. In contrast to acetylated histones, methylated histones located around TSSs are linked to both gene activation and repression (see Background). Furthermore, the functions of many methylated histones are still unknown. Therefore, for histone methylation, we made the following two groups, one having H3K4me1, -2 or -3, which are known to upregulate downstream genes, and the other having no modified histones. Distributions of gene expression levels were represented as box plots. *P *values for evaluating the significance of gene expression changes were calculated by the Wilcoxon rank sum test.

To compare the distributions of nucleosomes that had H3K4me3, were located upstream of TSSs (positions from -150 to -100 bp), and were associated with either broad promoters or peak promoters in cases where the downstream genes showed similar expression levels, we selected 1788 genes associated with broad promoters and 138 associated with peak promoters that had expression levels in the range of 250 to 750 (Additional File 4: Figure S4). The chi-squared test was applied to assess the difference in nucleosome distribution between these two types of promoter.

## Authors' contributions

TN performed all computational analyses. TN, NY, and RO designed the study and helped with the computational analyses. AK provided some data. RS supervised the research and helped to interpret the data. MT supervised the study. TN, NY, and RS wrote the paper. All authors read and approved the final manuscript.

## Supplementary Material

Additional file 1 **Supplemental figure 1**. Figure S1. Frequencies of occurrence of TATA boxes and CpG islands around transcription start sites (TSSs). Frequencies of the characteristic sequence patterns associated with promoters are shown by bar charts. The *y*-axis shows the proportions of broad and peak promoters that have TATA boxes (A) and CpG islands (B). Click here for file

Additional file 2 **Supplemental figure 2**. Figure S2. Distributions of Sp1 sites around transcription start sites (TSSs). Distributions of nucleosome regions and Sp1 sites around TSSs associated with broad (A) and peak (B) promoters are shown. Nucleosome position is defined as the center position of the nucleosome. The *x*-axis shows genomic positions with respect to TSSs (from -500 bp to 500 bp). Sp-1 sites were obtained by the FANTOM 4 project. Click here for file

Additional file 3 **Supplemental figure 3**. Figure S3. Distributions of the binding sites of two transcription factors (MAZ and PU.1) around transcription start sites (TSSs). Distributions of nucleosome regions and transcription factor binding sites around TSSs associated with broad (A: MAZ, C: PU.1) and peak (B: MAZ, D: PU.1) promoters are shown. Nucleosome position is defined as the center position of the nucleosome. The *x*-axis shows genomic positions with respect to TSSs (from -500 bp to 500 bp). Both MAZ and PU.1 sites were obtained by the FANTOM 4 project. Click here for file

Additional file 4 **Supplemental figure 4**. Figure S4. Distributions of expression levels of genes selected for comparison of broad and peak promoters associated with similar downstream gene expression. The box plots represent the distributions of the microarray intensities of the gene sets that were selected from among those associated with broad and peak promoters and that had similar expression levels (from 250 to 750). Click here for file

## References

[B1] Suzuki H Forrest AR van Nimwegen E Daub CO Balwierz PJ Irvine KM Lassmann T Ravasi T Hasegawa Y de Hoon MJ The transcriptional network that controls growth arrest and differentiation in a human myeloid leukemia cell line Nat Genet 2009 41 5 553 562 10.1038/ng.375 19377474 PMC6711855

[B2] Carninci P Kasukawa T Katayama S Gough J Frith MC Maeda N Oyama R Ravasi T Lenhard B Wells C The transcriptional landscape of the mammalian genome Science 2005 309 5740 1559 1563 16141072 10.1126/science.1112014

[B3] Barski A Cuddapah S Cui K Roh TY Schones DE Wang Z Wei G Chepelev I Zhao K High-resolution profiling of histone methylations in the human genome Cell 2007 129 4 823 837 10.1016/j.cell.2007.05.009 17512414

[B4] Schones DE Cui K Cuddapah S Roh TY Barski A Wang Z Wei G Zhao K Dynamic regulation of nucleosome positioning in the human genome Cell 2008 132 5 887 898 10.1016/j.cell.2008.02.022 18329373 PMC10894452

[B5] Wang Z Zang C Rosenfeld JA Schones DE Barski A Cuddapah S Cui K Roh TY Peng W Zhang MQ Combinatorial patterns of histone acetylations and methylations in the human genome Nat Genet 2008 40 7 897 903 10.1038/ng.154 18552846 PMC2769248

[B6] Shiraki T Kondo S Katayama S Waki K Kasukawa T Kawaji H Kodzius R Watahiki A Nakamura M Arakawa T Cap analysis gene expression for high-throughput analysis of transcriptional starting point and identification of promoter usage Proc Natl Acad Sci USA 2003 100 26 15776 15781 10.1073/pnas.2136655100 14663149 PMC307644

[B7] Plessy C Bertin N Takahashi H Simone R Salimullah M Lassmann T Vitezic M Severin J Olivarius S Lazarevic D Linking promoters to functional transcripts in small samples with nanoCAGE and CAGEscan Nat Methods 7 7 528 534 10.1038/nmeth.1470 PMC2906222 20543846

[B8] Carninci P Sandelin A Lenhard B Katayama S Shimokawa K Ponjavic J Semple CA Taylor MS Engstrom PG Frith MC Genome-wide analysis of mammalian promoter architecture and evolution Nat Genet 2006 38 6 626 635 10.1038/ng1789 16645617

[B9] Kratz A Arner E Saito R Kubosaki A Kawai J Suzuki H Carninci P Arakawa T Tomita M Hayashizaki Y Core promoter structure and genomic context reflect histone 3 lysine 9 acetylation patterns BMC Genomics 2010 11 257 10.1186/1471-2164-11-257 20409305 PMC2867832

[B10] Frith MC Valen E Krogh A Hayashizaki Y Carninci P Sandelin A A code for transcription initiation in mammalian genomes Genome Res 2008 18 1 1 12 18032727 10.1101/gr.6831208 PMC2134772

[B11] Kawaji H Frith MC Katayama S Sandelin A Kai C Kawai J Carninci P Hayashizaki Y Dynamic usage of transcription start sites within core promoters Genome Biol 2006 7 12 R118 10.1186/gb-2006-7-12-r118 17156492 PMC1794431

[B12] Ponjavic J Lenhard B Kai C Kawai J Carninci P Hayashizaki Y Sandelin A Transcriptional and structural impact of TATA-initiation site spacing in mammalian core promoters Genome Biol 2006 7 8 R78 10.1186/gb-2006-7-8-r78 16916456 PMC1779604

[B13] Coleman RA Pugh BF Evidence for functional binding and stable sliding of the TATA binding protein on nonspecific DNA J Biol Chem 1995 270 23 13850 13859 10.1074/jbc.270.23.13850 7775443

[B14] Davey CA Richmond TJ DNA-dependent divalent cation binding in the nucleosome core particle Proc Natl Acad Sci USA 2002 99 17 11169 11174 10.1073/pnas.172271399 12169666 PMC123228

[B15] Luger K Mader AW Richmond RK Sargent DF Richmond TJ Crystal structure of the nucleosome core particle at 2.8 A resolution Nature 1997 389 6648 251 260 10.1038/38444 9305837

[B16] Kornberg RD Lorch Y Twenty-five years of the nucleosome, fundamental particle of the eukaryote chromosome Cell 1999 98 3 285 294 10.1016/S0092-8674(00)81958-3 10458604

[B17] Lister R Pelizzola M Dowen RH Hawkins RD Hon G Tonti-Filippini J Nery JR Lee L Ye Z Ngo QM Human DNA methylomes at base resolution show widespread epigenomic differences Nature 2009 462 7271 315 322 10.1038/nature08514 19829295 PMC2857523

[B18] Wyrick JJ Holstege FC Jennings EG Causton HC Shore D Grunstein M Lander ES Young RA Chromosomal landscape of nucleosome-dependent gene expression and silencing in yeast Nature 1999 402 6760 418 421 10.1038/46567 10586882

[B19] Yuan GC Liu YJ Dion MF Slack MD Wu LF Altschuler SJ Rando OJ Genome-scale identification of nucleosome positions in S. cerevisiae Science 2005 309 5734 626 630 10.1126/science.1112178 15961632

[B20] Jiang C Pugh BF Nucleosome positioning and gene regulation: advances through genomics Nat Rev Genet 2009 10 3 161 172 19204718 10.1038/nrg2522 PMC4860946

[B21] Park PJ ChIP-seq: advantages and challenges of a maturing technology Nat Rev Genet 2009 10 10 669 680 10.1038/nrg2641 19736561 PMC3191340

[B22] Mavrich TN Ioshikhes IP Venters BJ Jiang C Tomsho LP Qi J Schuster SC Albert I Pugh BF A barrier nucleosome model for statistical positioning of nucleosomes throughout the yeast genome Genome Res 2008 18 7 1073 1083 10.1101/gr.078261.108 18550805 PMC2493396

[B23] Ozsolak F Song JS Liu XS Fisher DE High-throughput mapping of the chromatin structure of human promoters Nat Biotechnol 2007 25 2 244 248 10.1038/nbt1279 17220878

[B24] Smith AE Chronis C Christodoulakis M Orr SJ Lea NC Twine NA Bhinge A Mufti GJ Thomas NS Epigenetics of human T cells during the G0-- > G1 transition Genome Res 2009 19 8 1325 1337 10.1101/gr.085530.108 19546172 PMC2720178

[B25] Bernstein BE Kamal M Lindblad-Toh K Bekiranov S Bailey DK Huebert DJ McMahon S Karlsson EK Kulbokas EJ Gingeras TR Genomic maps and comparative analysis of histone modifications in human and mouse Cell 2005 120 2 169 181 10.1016/j.cell.2005.01.001 15680324

[B26] Roh TY Cuddapah S Cui K Zhao K The genomic landscape of histone modifications in human T cells Proc Natl Acad Sci USA 2006 103 43 15782 15787 10.1073/pnas.0607617103 17043231 PMC1613230

[B27] Vakoc CR Mandat SA Olenchock BA Blobel GA Histone H3 lysine 9 methylation and HP1gamma are associated with transcription elongation through mammalian chromatin Mol Cell 2005 19 3 381 391 10.1016/j.molcel.2005.06.011 16061184

[B28] Karlic R Chung HR Lasserre J Vlahovicek K Vingron M Histone modification levels are predictive for gene expression Proc Natl Acad Sci USA 2010 107 7 2926 2931 10.1073/pnas.0909344107 20133639 PMC2814872

[B29] Bannister AJ Miska EA Regulation of gene expression by transcription factor acetylation Cell Mol Life Sci 2000 57 8-9 1184 1192 11028911 10.1007/PL00000758 PMC11147133

[B30] Roopra A Qazi R Schoenike B Daley TJ Morrison JF Localized domains of G9a-mediated histone methylation are required for silencing of neuronal genes Mol Cell 2004 14 6 727 738 10.1016/j.molcel.2004.05.026 15200951

[B31] Ramirez-Carrozzi VR Braas D Bhatt DM Cheng CS Hong C Doty KR Black JC Hoffmann A Carey M Smale ST A unifying model for the selective regulation of inducible transcription by CpG islands and nucleosome remodeling Cell 2009 138 1 114 128 10.1016/j.cell.2009.04.020 19596239 PMC2712736

[B32] Tolstorukov MY Kharchenko PV Goldman JA Kingston RE Park PJ Comparative analysis of H2A.Z nucleosome organization in the human and yeast genomes Genome Res 2009 19 6 967 977 10.1101/gr.084830.108 19246569 PMC2694475

[B33] Albert I Mavrich TN Tomsho LP Qi J Zanton SJ Schuster SC Pugh BF Translational and rotational settings of H2A.Z nucleosomes across the Saccharomyces cerevisiae genome Nature 2007 446 7135 572 576 10.1038/nature05632 17392789

[B34] Jin C Zang C Wei G Cui K Peng W Zhao K Felsenfeld G H3.3/H2A.Z double variant-containing nucleosomes mark 'nucleosome-free regions' of active promoters and other regulatory regions Nat Genet 2009 41 8 941 945 10.1038/ng.409 19633671 PMC3125718

[B35] Bucher P Weight matrix descriptions of four eukaryotic RNA polymerase II promoter elements derived from 502 unrelated promoter sequences J Mol Biol 1990 212 4 563 578 10.1016/0022-2836(90)90223-9 2329577

[B36] Karolchik D Baertsch R Diekhans M Furey TS Hinrichs A Lu YT Roskin KM Schwartz M Sugnet CW Thomas DJ The UCSC Genome Browser Database Nucleic Acids Res 2003 31 1 51 54 10.1093/nar/gkg129 12519945 PMC165576

[B37] Butler JE Kadonaga JT The RNA polymerase II core promoter: a key component in the regulation of gene expression Genes Dev 2002 16 20 2583 2592 10.1101/gad.1026202 12381658

[B38] Ioshikhes IP Zhang MQ Large-scale human promoter mapping using CpG islands Nat Genet 2000 26 1 61 63 10.1038/79189 10973249

[B39] Wang J Hannenhalli S A mammalian promoter model links cis elements to genetic networks Biochem Biophys Res Commun 2006 347 1 166 177 10.1016/j.bbrc.2006.06.062 16806065

[B40] Bhandare R Schug J Le Lay J Fox A Smirnova O Liu C Naji A Kaestner KH Genome-wide analysis of histone modifications in human pancreatic islets Genome Res 20 4 428 433 10.1101/gr.102038.109 PMC2847745 20181961

[B41] Segal E Fondufe-Mittendorf Y Chen L Thastrom A Field Y Moore IK Wang JP Widom J A genomic code for nucleosome positioning Nature 2006 442 7104 772 778 10.1038/nature04979 16862119 PMC2623244

[B42] Cairns BR The logic of chromatin architecture and remodelling at promoters Nature 2009 461 7261 193 198 10.1038/nature08450 19741699

[B43] Illingworth RS Bird AP CpG islands--'a rough guide' FEBS Lett 2009 583 11 1713 1720 10.1016/j.febslet.2009.04.012 19376112

[B44] Lebrun A Shakked Z Lavery R Local DNA stretching mimics the distortion caused by the TATA box-binding protein Proc Natl Acad Sci USA 1997 94 7 2993 2998 10.1073/pnas.94.7.2993 9096334 PMC20310

[B45] Brayer KJ Segal DJ Keep your fingers off my DNA: protein-protein interactions mediated by C2H2 zinc finger domains Cell Biochem Biophys 2008 50 3 111 131 10.1007/s12013-008-9008-5 18253864

[B46] Tirosh I Barkai N Two strategies for gene regulation by promoter nucleosomes Genome Res 2008 18 7 1084 1091 10.1101/gr.076059.108 18448704 PMC2493397

[B47] Bock C Walter J Paulsen M Lengauer T CpG island mapping by epigenome prediction PLoS Comput Biol 2007 3 6 e110 10.1371/journal.pcbi.0030110 17559301 PMC1892605

[B48] Zhang Y Shin H Song JS Lei Y Liu XS Identifying positioned nucleosomes with epigenetic marks in human from ChIP-Seq BMC Genomics 2008 9 537 10.1186/1471-2164-9-537 19014516 PMC2596141

[B49] Sandelin A Alkema W Engstrom P Wasserman WW Lenhard B JASPAR: an open-access database for eukaryotic transcription factor binding profiles Nucleic Acids Res 2004 32 Database D91 94 10.1093/nar/gkh012 PMC308747 14681366

