# Supplementary figures and images for: Tight associations between transcription promoter type and epigenetic variation in histone positioning and modification
Source: BMC Genomics. 2011 Aug 17;12:416. doi: 10.1186/1471-2164-12-416 (PMC3170308; doi:10.1186/1471-2164-12-416)

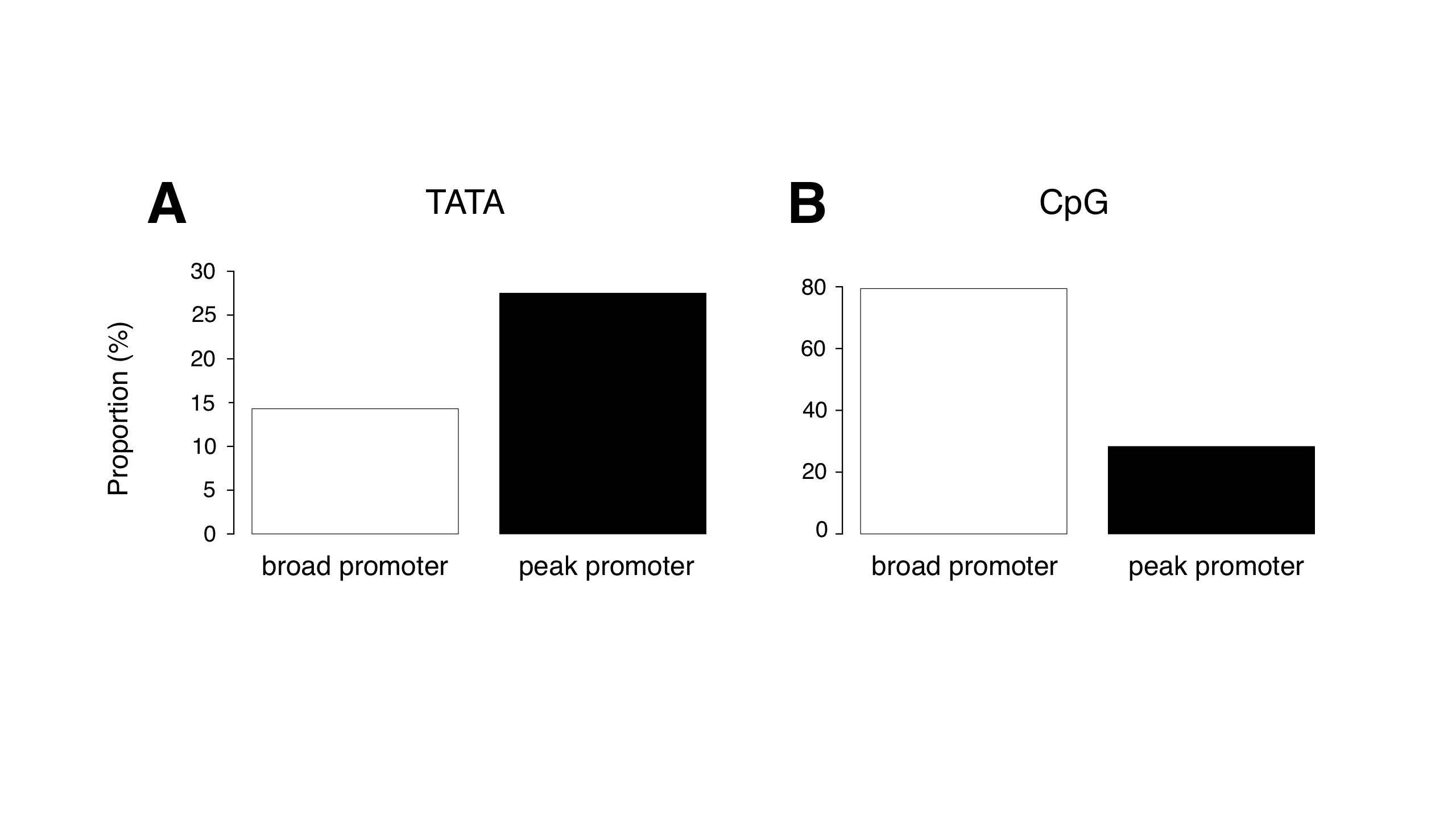

Supplement: Additional file 1 — Supplemental figure 1. Figure S1. Frequencies of occurrence of TATA boxes and CpG islands around transcription start sites (TSSs). Frequencies of the characteristic sequence patterns associated with promoters are shown by bar charts. The y-axis shows the proportions of broad and peak promoters that have TATA boxes (A) and CpG islands (B). [file 1471-2164-12-416-S1.JPEG]

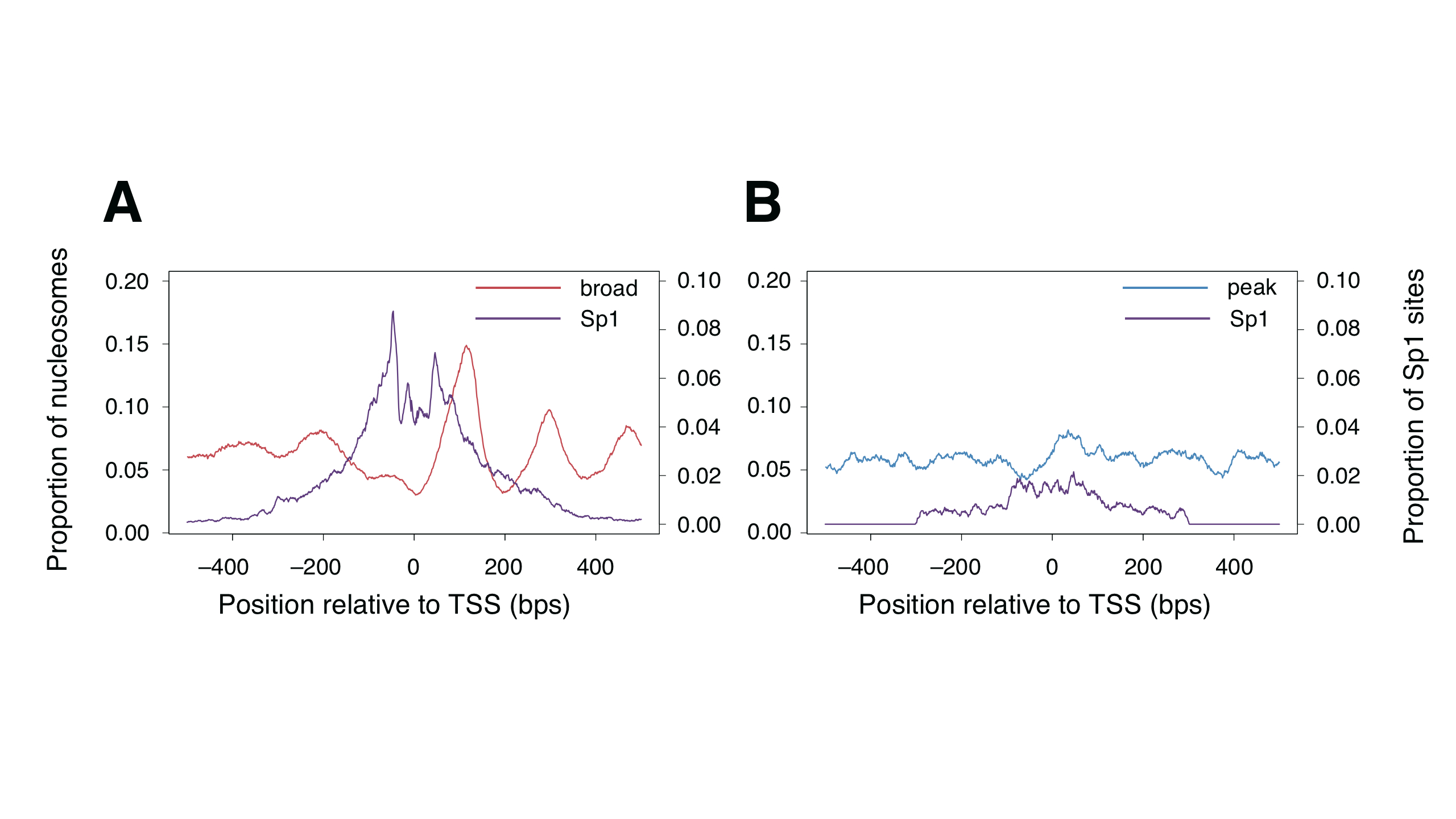

Supplement: Additional file 2 — Supplemental figure 2. Figure S2. Distributions of Sp1 sites around transcription start sites (TSSs). Distributions of nucleosome regions and Sp1 sites around TSSs associated with broad (A) and peak (B) promoters are shown. Nucleosome position is defined as the center position of the nucleosome. The x-axis shows genomic positions with respect to TSSs (from -500 bp to 500 bp). Sp-1 sites were obtained by the FANTOM 4 project. [file 1471-2164-12-416-S2.JPEG]

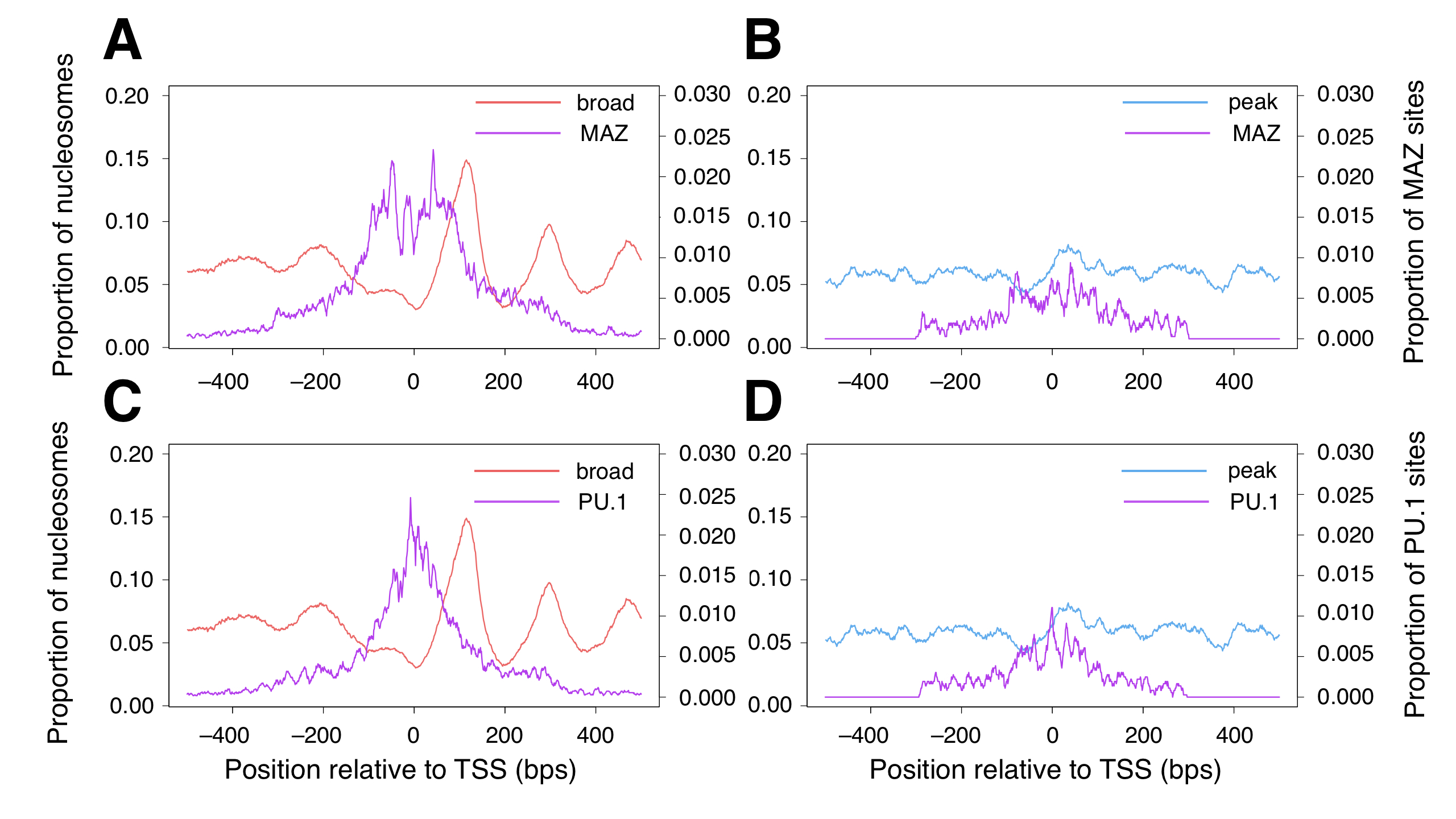

Supplement: Additional file 3 — Supplemental figure 3. Figure S3. Distributions of the binding sites of two transcription factors (MAZ and PU.1) around transcription start sites (TSSs). Distributions of nucleosome regions and transcription factor binding sites around TSSs associated with broad (A: MAZ, C: PU.1) and peak (B: MAZ, D: PU.1) promoters are shown. Nucleosome position is defined as the center position of the nucleosome. The x-axis shows genomic positions with respect to TSSs (from -500 bp to 500 bp). Both MAZ and PU.1 sites were obtained by the FANTOM 4 project. [file 1471-2164-12-416-S3.JPEG]

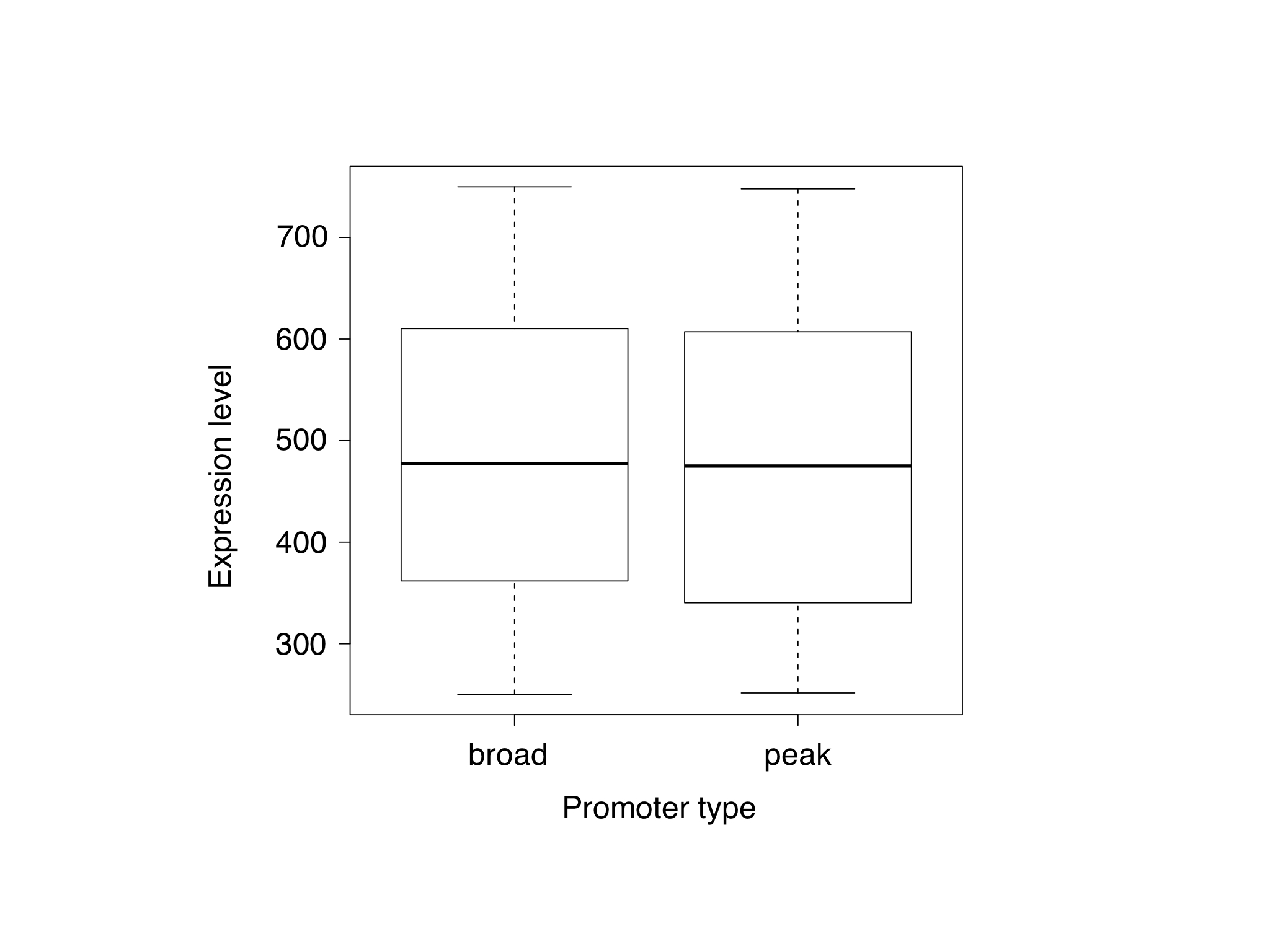

Supplement: Additional file 4 — Supplemental figure 4. Figure S4. Distributions of expression levels of genes selected for comparison of broad and peak promoters associated with similar downstream gene expression. The box plots represent the distributions of the microarray intensities of the gene sets that were selected from among those associated with broad and peak promoters and that had similar expression levels (from 250 to 750). [file 1471-2164-12-416-S4.JPEG]
